# Interim prostacyclin therapy for an isolated disconnected pulmonary artery: a case report

**DOI:** 10.1186/1752-1947-4-168

**Published:** 2010-06-02

**Authors:** Victor Grech, Cynthia Grixti

**Affiliations:** 1 Paediatric Department, Mater Dei Hospital, Disneyland, Tal-Qroqq, Malta

## Abstract

**Introduction:**

Disconnected pulmonary arteries are unusual and may result in pulmonary hypertension with acute right heart failure.

**Case presentation:**

We report a case of a three-month-old Asian girl who presented with heart failure and severe pulmonary hypertension due to a disconnected right pulmonary artery. An epoprostenol (prostacyclin) infusion was instrumental in lowering pulmonary artery pressures and stabilizing the child prior to surgery.

**Conclusions:**

This is, to the best of our knowledge, the first report of successful prostacyclin usage in such a situation.

## Introduction

Disconnected pulmonary arteries are unusual, and are almost invariably associated with conotruncal abnormalities [1]. We report a three-month-old infant who presented in heart failure and severe pulmonary hypertension due to a disconnected right pulmonary artery in the absence of conotruncal anomalies. An epoprostenol (prostacyclin) infusion was instrumental in lowering pulmonary artery pressures and stabilizing the child prior to surgery.

## Case presentation

Our patient was a three-month-old Asian girl of English nationality, born through normal vaginal delivery at full term to healthy and unrelated parents after an uneventful pregnancy. Her birth weight was 3.14 kg. She presented with tachypnoea, poor feeding and a cough. Examination showed irritability of the child, with respiratory distress, subcostal retraction and saturations of 80% to 85%, which improved marginally with nasal prong oxygen. She had 4 cm hepatomegaly and auscultation showed a 2/6 early and midsystolic murmur at the lower left sternal edge with a rather loud and single second sound. A chest X-ray showed cardiomegaly with bilateral pulmonary plethora.

**Figure 1:**
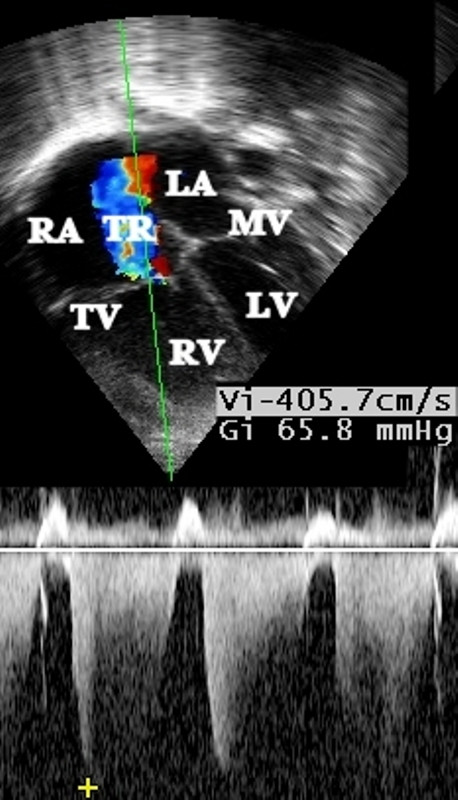
**Echocardiogram Doppler gradient in a four-chamber view demonstrating a gradient of 65 mmHg from right ventricle to right atrium as measured by the tricuspid regurgitation jet (LA: left atrium, MV: mitral valve, LV: Left ventricle, RA: right atrium, TV: tricuspid valve, RV: right ventricle, TR: tricuspid regurgitation jet)**.

**Figure 2:**
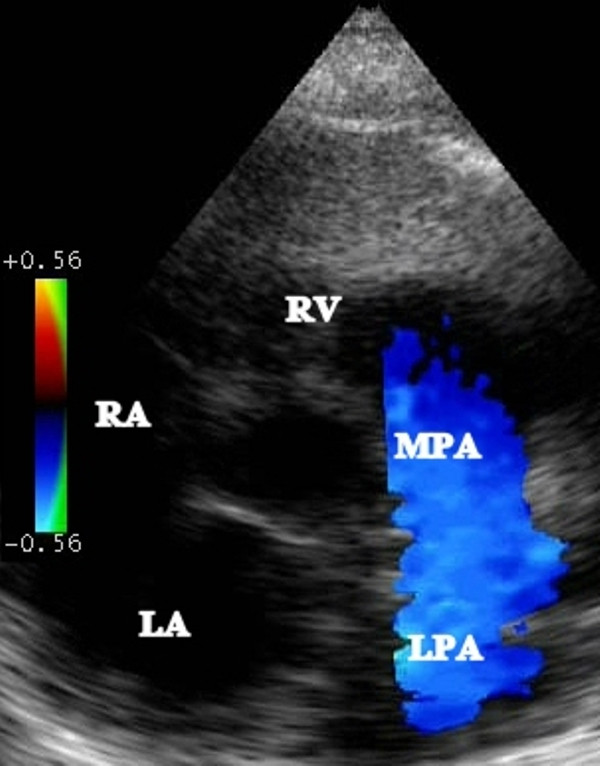
**Parasternal short-axis color Doppler view of the right ventricular outflow tract showing the absence of the right pulmonary artery (LA: left atrium, RA: right atrium, RV: right ventricle, MPA: main pulmonary artery, LPA: left pulmonary artery)**.

An echocardiogram showed an atrial septal defect with bidirectional flow and moderate tricuspid regurgitation with a peak gradient in the mid-60 s mmHg (Figure 1). The right pulmonary artery could not be visualized (Figure 2). An aortopulmonary collateral artery was not visualized at this stage, but a diagnosis of disconnected right pulmonary artery was made.

The infant began to sustain severe episodes of cyanosis which were relieved with an epoprostenol infusion. Pulmonary artery pressures also fell as evinced by the tricuspid regurgitation gradient, which fell to the 20 s in mmHg on echocardiographic estimation.

After the transfer of the patient to a tertiary centre, catheterization demonstrated bilateral ductal stumps but had no flow to any vessels. A large leash of vessels was identified, supplying right thoracic, right internal mammary and right subclavian arteries toward the right hilum. No proximal right pulmonary artery segment could be demonstrated, and a distal pulmonary artery was faintly visible at the level of the hilum. During surgery, an aortopulmonary collateral artery supplying the right lung was identified and the infant underwent uneventful pulmonary artery reconstruction using an 8 mm Goretex graft. The baby had been on epoprostenol for a total of nine days. She had been reviewed regularly for six months since her procedure and remained well, off treatment and not in heart failure. The auscultatory findings were completely normal and an echocardiography showed a normal flow pattern into the right pulmonary artery with no turbulence and a velocity of 1.3 m/s. The family have since emigrated from the country.

## Discussion

The disconnection of a pulmonary artery is rare and may be difficult to diagnose echocardiographically [2], and duct-dependent pulmonary arteries may require a prostaglandin infusion for a diagnosis to be elicited [3]. This condition is almost invariably associated with conotruncal anomalies and treatment is surgical [4]. A large series of 108 cases had shown that the right pulmonary artery is more commonly involved than the left, with the former being more commonly associated with patent arterial duct and aortopulmonary window, and the latter being more commonly associated with conotruncal defects and aortic arch abnormalities [5].

Our patient was in an unusual situation, in that this anomaly was not associated with conotruncal anomalies [1], as described in association with fetal valproate syndrome [6]. Moreover, our patient responded well to an epoprostenol infusion. Epoprostenol is a pulmonary vasodilator. By lowering the pulmonary vascular resistance in the remaining lung that was connected to the right ventricle, the right ventricle of the patient recovered its function and this allowed the baby to survive until the transfer to a tertiary centre for surgery. Epoprostenol may be used for pulmonary hypertension of any aetiology [7], and indeed, there are a variety of pulmonary vasodilators that may be used in these settings. These may be administered through a variety of routes, such as intravenously (as in our patient), orally, through inhalation, or even as part of a gas mixture in ventilated patients [8]. Epoprostenol was used in our patient because of the simplicity of its use (simple intravenous infusion). This is a crucial issue when patients must be transferred. And, as in our case, the transfer involved an ambulance trip to the airport, an airplane flight and another ambulance trip.

## Conclusions

Our patient, who suffered right heart failure and severe pulmonary hypertension, stabilized with the help of an epoprostenol infusion and was safely transferred for treatment at a tertiary centre. This is an interim method of treatment that has not yet been documented, to the best of our knowledge.

## Consent

Written informed consent was obtained from the patient's next-of-kin for publication of this case report and any accompanying images. A copy of the written consent is available for review by the Editor-in-Chief of this journal.

## Competing interests

The authors declare that they have no competing interests.

## Authors' contributions

Both authors contributed equally to the creation of this manuscript. VG supervised the case and performed the echocardiography, while CG wrote the first draft of the paper and helped with the literature search. Both authors read and approved the final manuscript.

## References

[B1] Vida VL Sanders SP Bottio T Maschietto N Rubino M Milanesi O Stellin G Anomalous origin of one pulmonary artery from the ascending aorta Cardiol Young 2005 15 176 181 10.1017/S1047951105000363 15845161

[B2] Kim TK Choe YH Kim HS Ko JK Lee YT Lee HJ Park JH Anomalous origin of the right pulmonary artery from the ascending aorta: diagnosis by magnetic resonance imaging Cardiovasc Intervent Radiol 1995 18 118 121 10.1007/BF02807236 7773994

[B3] Patel JN Lantin-Hermoso MR Utility of prostaglandin in the identification of discontinuous pulmonary arteries by echocardiography Pediatr Cardiol 2003 24 595 597 10.1007/s00246-003-0528-x 12881776

[B4] Murphy DN Winlaw DS Cooper SG Nunn GR Successful early surgical recruitment of the congenitally disconnected pulmonary artery Ann Thorac Surg 2004 77 29 35 10.1016/S0003-4975(03)01504-2 14726029

[B5] Kutsche LM Van Mierop LH Anomalous origin of a pulmonary artery from the ascending aorta: associated anomalies and pathogenesis Am J Cardiol 1988 61 850 856 10.1016/0002-9149(88)91078-8 3354450

[B6] Mo CN Ladusans EJ Anomalous right pulmonary artery origins in association with the fetal valproate syndrome J Med Genet 1999 36 83 84 9950375 PMC1762948

[B7] Kao B Balzer DT Huddleston CB Canter CE Long-term prostacyclin infusion to reduce pulmonary hypertension in a pediatric cardiac transplant candidate prior to transplantation J Heart Lung Transplant 2001 20 785 788 10.1016/S1053-2498(01)00231-5 11448812

[B8] Gomberg-Maitland M Olschewski H Prostacyclin therapies for the treatment of pulmonary arterial hypertension Eur Respir J 2008 31 891 901 10.1183/09031936.00097107 18378784

